# The effectiveness of a longitudinal ultrasound curriculum for general pediatricians working in a Puerto Rican emergency department: a pilot study

**DOI:** 10.1186/s13089-020-00169-4

**Published:** 2020-04-21

**Authors:** Veronica Sepulveda-Ortiz, Fred Warkentine, Rebecca Starr-Seal, Anna Rominger

**Affiliations:** Division of Pediatric Emergency Medicine, Department of Pediatrics, University of Louisville, 571 S. Floyd Street, Suite 412, Louisville, KY 40202 USA

**Keywords:** Point-of-care ultrasound, POCUS, Pediatric emergency medicine, PoCUS education, Resource-limited settings

## Abstract

**Background:**

The Hospital Pediatrico Universitario (HOPU) is the principal institution in Puerto Rico offering medical services to the children of the island and the Caribbean. There is limited use of point-of-care ultrasound (PoCUS) in their emergency department (ED) and obtaining an ultrasound through radiology is prohibitively time consuming. The objective of this study is to increase PoCUS knowledge and comfort by the ED physicians in the HOPU pediatric emergency department.

**Results:**

Thirteen general pediatricians completed the entire PoCUS course, but only 10 completed both the pre- and post-tests and therefore included in the analysis (*N* = 10). Pretest scores ranged from 30 to 63.3% with a mean of 41.6% [standard deviation (SD) 9.95]. The posttest scores ranged from 55 to 96%, with a mean of 66.1% (SD 12.26). The mean difference in scores was 24.42% (95% confidence interval 17.9, 30.9) with a significance of *p* < 0.05 and range of 8.3–36.6%. Physician experience and confidence with each topic improved from baseline. After the course, the majority of the participants (> 70%) had at least some confidence in 5 of the 6 topics.

**Conclusions:**

In EDs with limited resources, a longitudinal PoCUS educational curriculum is effective in improving the knowledge and comfort of physicians with limited PoCUS experience. The effectiveness of scheduled, repeated courses to refresh and refocus participants was highlighted following the unexpected challenges encountered during the course, including multiple natural disasters.

## Background

PoCUS is an affordable, safe, and efficient imaging modality with a wide variety of uses in the medical field, particularly in the emergency setting. Medical providers in Europe have utilized ultrasound for decades and over the last 20 years; its use has exploded in the United States. A PoCUS examination is performed at bedside with immediate results, without radiation exposure to the patient or provider. In addition, ultrasonography is a fraction of the cost of other imaging modalities, including radiography, computed tomography (CT), and magnetic resonance imaging (MRI) which makes it a more accessible imaging option in financially strained circumstances.

Since 1985, the World Health Organization (WHO) has recommended ultrasonography for developing countries because it is portable, inexpensive, non-invasive, safe, and provides information immediately [6, 8]. Via et al. noted that point-of-care ultrasound (PoCUS) has a significant impact in undifferentiated shock, narrowing the differential diagnosis and improving the specificity of early recognition of hypovolemia and septic shock cardiovascular patterns [8]. Because of this, the authors suggest that PoCUS should have an integral role in management of unstable patients in developing countries [8]. In addition, lung PoCUS is very effective for the early diagnosis of various infectious respiratory conditions, which is a leading cause of childhood mortality in developing countries [8, 9]. Short-term medical mission trips to resource-limited regions have been using PoCUS for many years. However, the best strategy to make a lasting change in these areas is to equip local providers with ultrasound machines and the knowledge to use them so they can continuously provide a higher level of care to the local people.

Short-term PoCUS training programs are proven to deliver adequate knowledge and skills to novices [8]. Shah et al. conducted an educational intervention in Rwanda to create an effective PoCUS curriculum for inpatient providers at two of the local hospitals [6]. They concluded that PoCUS is a very teachable skill with an intensive training course and hands-on teaching [6]. They introduced a 9-week course with 1-h lectures three times per week for the first 3 weeks and then one time per week for the remaining 6 weeks with 1–2 h of guided scanning practice following each lecture [6, 7]. The most common clinical practice change noted after the course was a new plan to perform a surgical procedure following the results of the PoCUS [7]. The investigators found other types of changes in the patient care plan based on PoCUS results, including medication changes, referrals to a specialty clinic, cancellation of surgical procedures, and referrals for further radiologic evaluation with CT scanning [7].

More recently, Rominger et al. implemented a 12-month longitudinal PoCUS curriculum for primary care physicians working in rural outpatient clinics in Mexico [5]. They organized 4-day PoCUS teaching sessions each separated by approximately 3–4 months [5]. By having recurring short-term trips, the training did not interrupt the regular schedule of the local physicians and was more feasible for educators traveling to the area. The sessions included lectures and hands-on training focused on different topics at each session. They found that the use of PoCUS changed the patient diagnosis and clinical management in about 1/3 of cases [5]. They demonstrated that bedside PocUS education programs targeted to local physicians in outpatient settings is an effective strategy to equip them with a tool to improve the clinical management of their patients.

The effectiveness of a PoCUS curriculum is not limited to developing countries. A study by Clay et al. focused on United States (US) Internal Medicine residents, found that a single day of intensive PoCUS training at the beginning of the year yielded significant improvement in PoCUS interpretation skills [1, 2]. They used a 30-question assessment to measure bedside PoCUS knowledge prior to, immediately post, and 6 months post-training. Assessment performance increased by almost 25% and remained significantly better at 6 months [1, 2]. Noble et al. also performed an educational study directed towards United States (US) Emergency Medicine physicians and interns [4]. The participants of this study received an “introduction to PoCUS” course. An assessment before and 6 months after the introductory course was administered using a standardized image-based ultrasound test. There was a significant improvement in PoCUS knowledge for both faculty and interns, which also persisted for 6 months following the introductory course [4].

The Hospital Pediatrico Universitario (HOPU) is the principal institution in Puerto Rico offering medical services to the children of Puerto Rico and the Caribbean. HOPU is the pediatric academic institution for the University of Puerto Rico School of Medicine and the clinical center for the pediatric residency program. It is the only pediatric hospital to provide tertiary services to the island and guarantees that its services are accessible to children of all socio-economic levels, including the medically indigent. The Emergency Department (ED) at HOPU receives about 10,000 pediatric patients annually. It is generally a low-flow but high-acuity ED where general pediatricians and residents provide health care 24 h a day, 7 days a week. Prior to this study, there were no ultrasound machines in the Emergency Department and the physicians had no PoCUS training. Access to ultrasound was available through the Radiology Department but since the Radiology Department serves the entire Medical Center and not just the pediatric hospital, obtaining an official ultrasound report takes 24–48 h. Many of the conditions seen in the Emergency Department are easily identified and managed with the appropriate use of PoCUS. For example, the identification of cellulitis, abscess, free fluid in the abdomen (traumatic or nontraumatic), hydronephrosis, pericardial effusion, pleural effusion, appendicitis, intussusception, and global heart function. If the providers have basic PoCUS knowledge and skills, it would greatly aid in the local management of many medical conditions, reduce the number of referrals for imaging, and expedite the care of patients who do have a serious medical or surgical condition.

The objective of this study is to increase ultrasound knowledge and comfort by the ED physicians in the HOPU pediatric emergency department. Although Puerto Rico is a US territory, its pediatric health care model and funding can be likened to a resource-limited site with the closest additional pediatric resources located 1000 miles away in Miami, Florida. Therefore, it is a unique setting which cannot be simply grouped with US facilities or resource-limited ones, so many of the previous studies may not be easily generalizable to this particular institution. Therefore, the authors sought to identify an effective ultrasound curriculum for the HOPU ED providers.

## Methods

This is a longitudinal educational curriculum conducted at the HOPU in San Juan, Puerto Rico. It is a pilot study to determine if a longitudinal approach would be an effective teaching strategy in this particular group of physicians. The instructors for the educational sessions included two pediatric emergency medicine (PEM) physicians, an emergency medicine (EM) physician and ultrasound fellow, and a PEM fellow from the University of Louisville. A PEM physician and ultrasound fellow from Indiana University joined the primary investigator on one of the trips. Both the University of Louisville and the University of Puerto Rico Institutional Review Boards reviewed this study and deemed it exempt.

Prior to initiation of the course work, the primary investigator conducted a needs assessment at HOPU with local physicians to determine the most relevant topics and commonly encountered regional pathologies. They designed the curriculum based on the findings in the needs assessment and desires of the local providers with a focus on trauma evaluation (FAST), bedsides cardiac evaluation for global function, identification of skin and soft tissue infections, and abdominal pathologies, specifically intussusception and appendicitis. To accommodate the schedules of the local physicians, the team taught the course in multiple sessions rather than one long conference, which the Puerto Rican providers preferred. In addition, the amount of information presented in one long conference can be overwhelming for novices and the investigators had concerns for adequate knowledge retention in a single course. The longitudinal approach allowed participants to master several PoCUS concepts before moving on to new ones. Lastly, this design put the educators at the site for multiple points in time to review previous concepts, answer additional questions, discuss images and cases, and review technique. The HOPU ED did not have an ultrasound machine in their department prior to the course; however, Norton Children’s Hospital donated a Sonosite M-turbo ultrasound machine with low-frequency (curvilinear), phased-array, and high-frequency (linear) probes.

The curriculum was designed based on the previous work by Rominger et al. in Mexico. This structure was used due to the time constraints of the local providers and the fact that the providers were all general pediatricians without subspecialty training, similar to the general practitioners trained in Mexico. The curriculum consisted of three, 2-day sessions given over 12 months. The instructors taught the material in English and, as a US territory, all participants were fluent in English. The first day consisted of lectures with hands-on training on healthy volunteers. The class ratio was 5–7 learners per two instructors. The second day of the course focused on hands-on examination of patients with pathologic findings on ultrasound. Prior to the practice sessions, the instructors identified patients with relevant pathology throughout the hospital and obtained consent (and assents as applicable) to ultrasound for learning purposes.

The participants had all completed a 3-year general pediatrics residency and were working in the HOPU ED as independent providers and supervising physicians. The investigators tested the baseline ultrasound knowledge of each participant prior to the first session using a 25-question written test. The test was developed as part of the PEM PoCUS credentialing program at the University of Louisville used to demonstrate bedside PoCUS competency. The primary investigator distributed the same test to participants 3 months after the final session to assess ultrasound knowledge. The results of the test were withheld from participants until the completion of the post-curriculum evaluation. They each completed a questionnaire prior to each session and again 3 months following the last session, which assessed their subjective experience and confidence in the various PoCUS modalities. The first teaching course was in August 2017 and focused on care and use of the ultrasound machine, introduction to PoCUS, focused assessment with sonography in trauma (FAST), and basic bedside cardiac ultrasound. The next session was scheduled for November 2017, but the curriculum was adjusted following multiple natural disasters in Puerto Rico during the fall of 2017. The next session moved to February 2018 and it included a half-day review of the previous information plus the introduction of lung, skin and soft tissue, kidney, bladder, and ultrasound-guided procedures. The final session was May 2018 and focused on cardiovascular, abdominal, and ocular ultrasound. The instructors uploaded the presentations from each session onto a google drive that the participants had access to for their reference and review. The participants continued to order formal ultrasound examinations on patients through the HOPU radiology department throughout the study following their own bedside PoCUS and assessment. This was done for the safety of the patients until the providers could show competency with ultrasound by conducting at least 30 scans for each body area and completing a practical assessment of their skills.

There was a PoCUS log with the machine to document the type of study conducted, reason for the imaging, presumed diagnosis, and querying if the PoCUS findings confirmed or changed the presumed diagnosis for the patient. Information included the provider’s initials, patient age, patient gender, and date of the study. This would allow the investigators to track the number of different PoCUS studies conducted and the number performed by each provider. This would allow the investigators to track the number of each type of study conducted by each participant. It would also allow investigators to determine if improved knowledge and experience with PoCUS was assisting diagnosis and treatment plans, specifically the clinical integration of the information. Participants downloaded de-identified images to a secure flash drive, which the senior investigator, who is a core instructor for the ultrasound curriculum at the University of Louisville and instructor at multiple national and international PoCUS courses, reviewed for technique, quality, and findings.

The investigators analyzed the basic demographic data for the participants and compared each one’s change in confidence and experience from the beginning to the end of the yearlong curriculum. Investigators compared the pretest and posttest averages using a paired T test. They conducted all analyses in IBM SPSS statistics version 24.

## Results

**Fig. 1:**
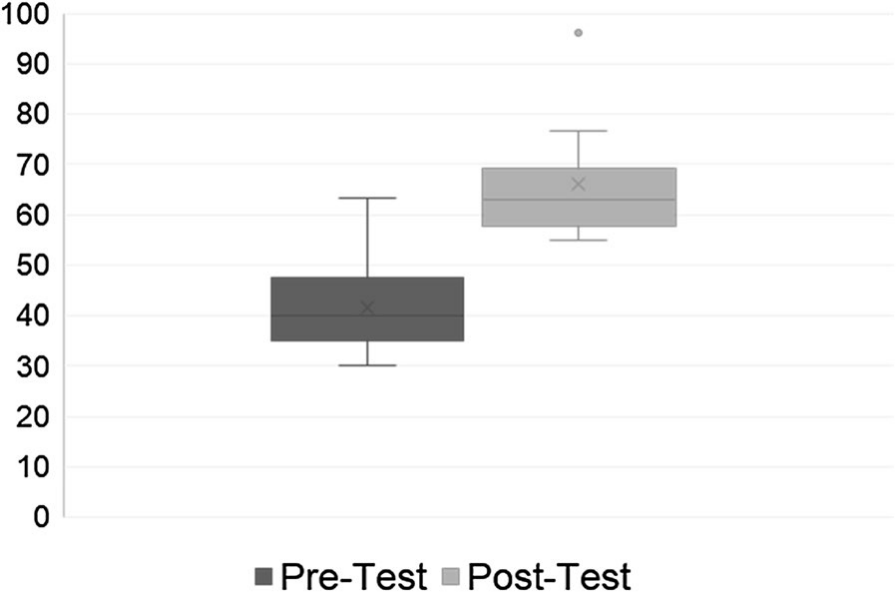
Pre- and post-intervention scores of participants in the ultrasound course

Ten physicians participated in all three PoCUS sessions and completed both the pretest and posttest. The posttest was distributed 3 months following the last PoCUS course. The pretest average score was 41.67% with a range of 30–63.3% and a standard deviation of 9.95 (Fig. 1). The posttest average was 66.1% with a range of 55–96% and a standard deviation of 12.26. The mean change in test score for the participants was 24.42% with a range of 8.3–36.6% and a standard deviation of 9.03 (*p* value < 0.01) (Fig. 1). Of note, this is meant to be a challenging test with a passing score of 75% for University of Louisville PEM physicians who are seeking bedside PoCUS credentialing.

**Fig. 2:**
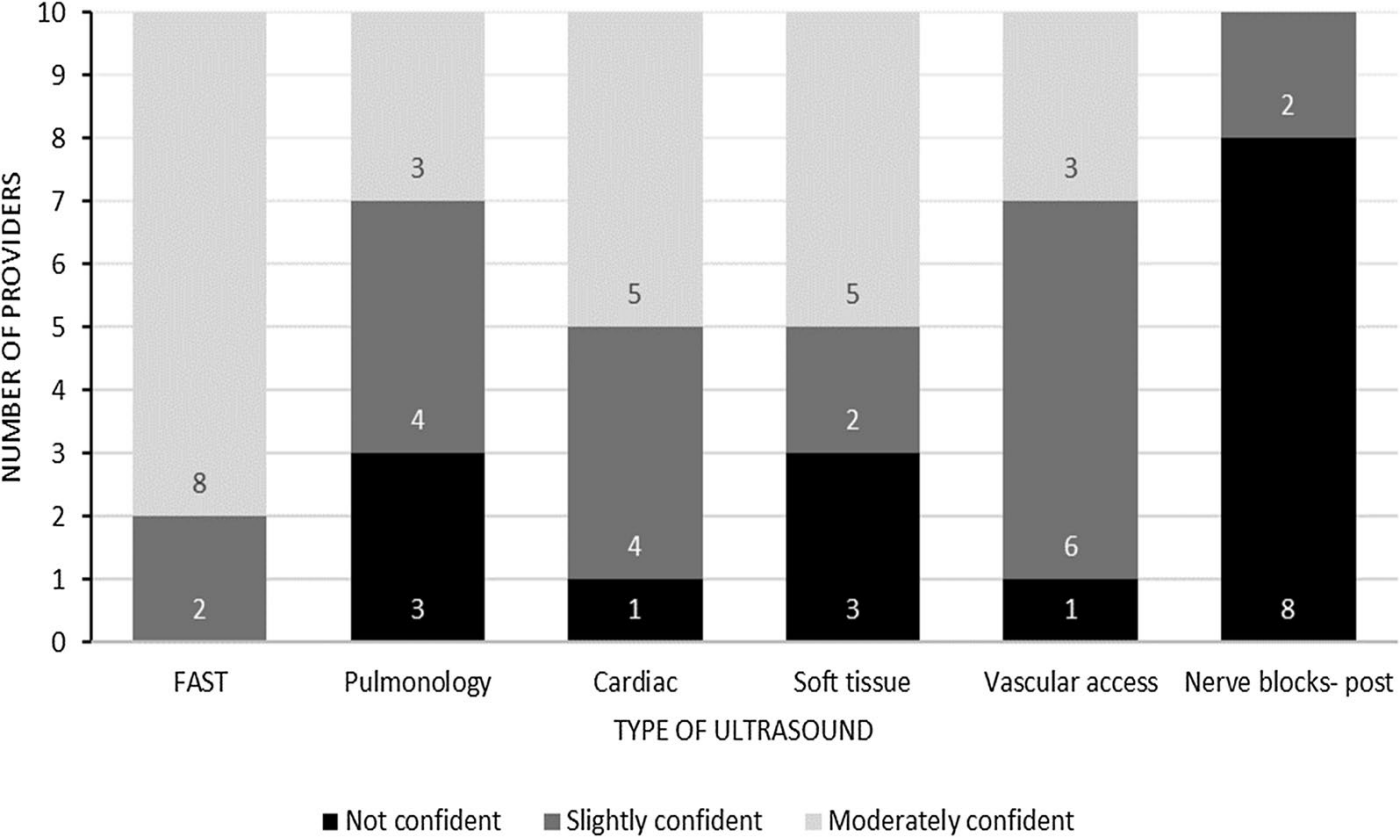
Provider confidence in ultrasonography of the various types of studies

Prior to the course, none of the participants expressed any confidence in their PoCUS examinations for FAST, lung, bedside cardiac, or nerve examination. “No confidence” was defined as no expectation of one’s ability to obtain and interpret the appropriate PoCUS examination. “Little confidence” was defined as an expectation in one’s abilities to obtain and correctly interpret the PoCUS image less than 50% of the time. “Moderate confidence” was defined as an expectation in one’s abilities to obtain and correctly interpret the PoCUS image 50–90% of the time. Lastly, “very confident” was defined as an expectation in one’s ability to obtain and correctly interpret the PoCUS image 90–100% of the time. There were nine of ten participants who had no confidence in their soft tissue and vascular access PoCUS examinations. Three months following the completion of the course, 80% were at least moderately confident with their FAST examinations and 50% were at least moderately confident with their cardiac and soft tissue examinations (Fig. 2). There were 70% of participants who had some confidence in their lung and 90% who had some confidence in their vascular access skills by the completion of the course (Fig. 2).

**Fig. 3:**
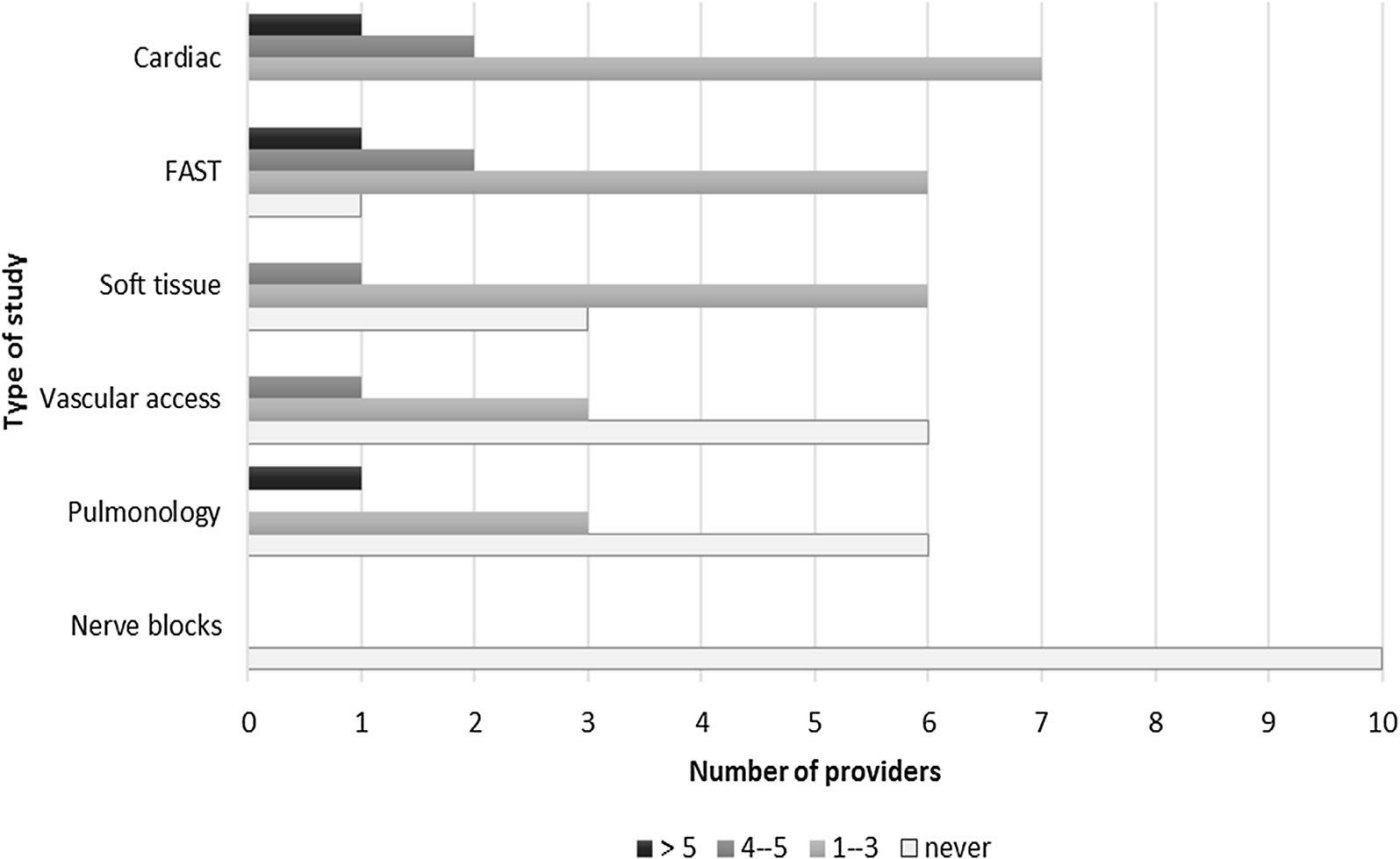
The frequency of each type of ultrasound conducted throughout the ultrasound course

Prior to the PoCUS course, 100% of participants had no experience with FAST, bedside cardiac, lung, and nerve blocks. There were 90% of participants who had no experience with soft tissue and 80% had no experience with vascular access PoCUS. With the exception of nerve blocks, the number of participants with experience in each of the studies increased following the course (Fig. 3).

**Fig. 4:**
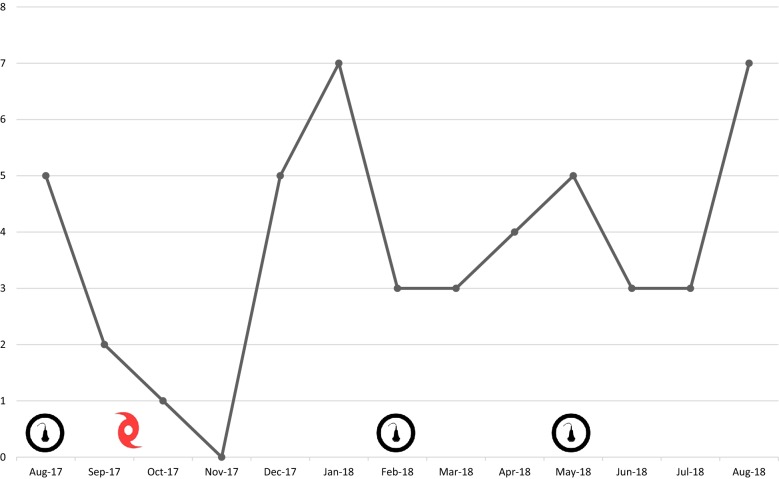
The number of studies saved on the ultrasound machine over the 12-month curriculum. The images of the ultrasound probe correspond to each of the three teaching sessions. The hurricane symbol corresponds to the timing of the two hurricanes that devastated the island

There were 52 PoCUS studies saved on the ultrasound machine over the course of the curriculum (Fig. 4). The number of studies conducted had a sharp drop during the months of September, October, and November 2017 where there were only three recorded studies for all 3 months. This coincides with the devastation experienced on the island following multiple significant natural disasters. The providers recorded the most scans in August 2018, which is 12 months after the initiation of the educational curriculum and 3 months following the final session. The most commonly completed PoCUS studies were bedside cardiac (36.5%) and FAST (15.3%) (Table 1). The providers did not keep an accurate ultrasound log and for many months following the hurricane because the log was displaced from its position on the stand with the ultrasound machine. Therefore, it was impossible to determine the exact number of body scans conducted by each provider to determine if anyone was eligible for a practical ultrasound assessment for independent scanning.

**Table 1:** The number and type of PoCUS saved and recorded on the ultrasound machine

Type of study	Number of PoCUS	Percentage (%)
Cardiac	19	36.5
FAST	8	15.3
Renal/bladder	6	11.5
Lung	5	9.6
Skin/soft tissue	4	7.7
Eye	3	5.8
Aorta/IVC	2	3.8
Other	5	9.6

Ninety-four percent of the scans were reviewed for image quality. There were 3 scans that were not saved correctly and the images could not be reviewed. Of the images reviewed, 69% had good image quality and could be easily interpreted by the investigators. Good image quality was defined as an image that clearly identifies the organ or area of concern with appropriate scanning through its entirety. For example, a good-quality image of an abscess or cellulitis had a scan from the area of normal skin and soft tissue through the abnormal area back to the area of normal skin and soft tissue on the other side. It also had the appropriate amount of depth where the area of concern or question is in the center of the screen. A good-quality image would show the appropriate orientation of the probe based in the screen indicator and image (i.e., image is not backwards or upside down), have the appropriate probe and the appropriate windows. For example, an adequate bedside cardiac PoCUS would have the correct probe, orientation and windows for a parasternal long axis, parasternal short axis, subxiphoid, and/or apical 4 chamber views with at least 3 of the traditional cardiac windows.

## Discussion

This study met the objective to increase the PoCUS knowledge of the physicians working in the HOPU pediatric ED. The participants’ scores increased by an average of almost 25%. This is similar to the improvement in immediate posttest scores following a single day of intensive training demonstrated by Clay [1, 2]; however, their participants’ average scores waned to an 18% increase 6 months following the initial intervention and may have decreased further if measured 12 months after the training. Noble showed a similar improvement of about 20% in ultrasound recognition scores after two back-to-back educational sessions and hands-on training [4]. Noble and Clay had the majority of the teaching sessions in a 1- or 2-day intense session with either no or optional teaching over the subsequent 6 months prior to repeat testing [1, 4]. Although both studies demonstrated improved PoCUS knowledge, Clay’s study suggests that some of this knowledge wanes with time. The investigators obtained the Puerto Rican physicians’ posttest scores 12 months after the initial intervention showing longer lasting knowledge retention of these initial concepts. This supports the notion that repeated short sessions has similar and somewhat improved long-term knowledge retention of basic ultrasound concepts compared to single, intense short-length interventions.

This curriculum consisted of 15 classroom/lecture hours, 15 required hands-on, and 10 optional extra hands-on hours over the course of the year, which is similar to the total training hours in the 9-week educational intervention in Rwanda [7]. The longitudinal approach is more feasible compared to Shah’s study in settings where educators cannot be in one location for 9 weeks teaching PoCUS. Repeated short sessions are more practical for both presenters and participants who are balancing the course with their regular clinical responsibilities at their respective home sites. A recent educational curriculum conducted in Mexico shows the effectiveness of a longitudinal teaching design in an outpatient, resource-limited setting [5]. Although Puerto Rico is not considered a true “resource limited” site, the investigators demonstrate that this same design can be effectively applied to various settings.

The HOPU participants had little to no confidence in bedside PoCUS. Following the educational intervention, the majority of participants self-report at least some confidence and many had moderate confidence with five of the six imaging modalities presented over the course. The participants all conducted at least 5 of each type of body scan during the educational sessions and hands-on training. Keddis et al. also evaluated the confidence level in a similar 5-point Likert scale and showed a comparable improvement in confidence immediately following short-term PoCUS teaching sessions [3]. However, Keddis did not evaluate the long-term retention of confidence in POCUS, which the investigators demonstrate in this intervention with participant self-reporting 12 months following initial educational session [3].

The HOPU ED providers were novice ultrasonographers with little to no experience with PoCUS prior to the educational intervention. The initial design called for ultrasound logs to accurately document the total number, type, and intent of studies for each participant. However, due to multiple natural disasters, which occurred on the island shortly after the initiation of the curriculum, the logs were not accurately documented. Without accurate documentation of number of scans per provider, no providers were eligible for a practical ultrasound evaluation. The pilot phase of the study showed acquisition of knowledge and confidence with this type of training, so the next phase will focus on documentation and credentialing. The primary investigator will become the local ultrasound champion in the HOPU ED at the completion of her fellowship. With a local champion, the ultrasound logs will be more accurate and she can conduct practical ultrasound assessments of the local physicians when they have completed at least 30 scans of a particular body area.

There were 52 studies saved on the ultrasound machine which were used in the analysis. At the completion of the curriculum, the participants report conducting between 58 and 131 PoCUS studies. This discrepancy suggests that the HOPU physicians were not saving all of their images and using PoCUS more than documented. The number of overall PoCUS studies is lower than those documented in previous interventions by Rominger and Shah [5, 7]; however, there were 3 months during the 12-month intervention when PoCUS were unable to be obtained due to power outages and physical limitations following the natural disasters. In addition, the previous studies were not limited to pediatrics and between 1/3 and 1/2 of their studies were obstetric, which was not included in this curriculum [5, 7]. The most common PoCUS study was abdomen and cardiac which was consistent with previous studies when the obstetric studies were removed. In the next phase of the study, the local champion will encourage the providers to save the images and can cross check the ultrasound logs to the saved images weekly.

The intervention required curriculum modification following two major natural disasters (Hurricanes Irma and Maria), which affected the island in September 2017 after the first educational session. The hospital was with limited power and resources for 3 months following the hurricanes. The second session was originally scheduled for November 2017, but because power and communication had not been consistently restored, the session was moved to February 2018. Because there was a prolonged time between educational sessions, the educators gave a review of the August information before the scheduled February session. There was a significant drop in PoCUS use from September 2017 through November 2017, but once there was restoration of consistent power and communication, there was a dramatic increase in PoCUS use. It can be theorized that the providers utilized PoCUS as an effective diagnostic modality when there were still limited resources while the island was recovering from the hurricanes. This supports the usefulness of PoCUS training in this setting and the value of a longitudinal curriculum. Having a scheduled session following the disaster recovery period allowed the learners to review the previous information after a period of limited use. In addition, almost 25% of initial participants did not complete the full 12-month curriculum and their information was removed from the study. These providers left the island permanently following the natural disasters.

### Limitations

The biggest limitation in this study was natural disasters, which resulted in loss of participants, decreased scanning months and adjustment of the originally scheduled curriculum. This also affected the documentation of images on the ultrasound logs which were used to track the number and type of study and the clinical applicability of the information. Upon discussion with the providers, it seemed that they were using the ultrasound machine much more frequently than they were documenting; however, only the documented studies could be used in analysis. This was beyond the control of the investigators; however, the remaining participants still showed increase in PoCUS knowledge, confidence and experience despite the circumstances. The total number of PoCUS studies and confidence level may have been affected by recall bias since each participant was asked to self-report this information at the completion of the course. The participants did not keep accurate ultrasound logs so the information was limited to subjective recall. In addition, the biggest limitation was no local ultrasound champion to oversee and answer real-time questions. This would have been helpful following the hurricanes when communication with providers on the island was limited.

## Conclusions

The investigators show that a longitudinal PoCUS curriculum is an effective design to teach PoCUS to local physicians working in acute ED settings. Due to unforeseen circumstances, 6 months of the intervention occurred during a resource-limited time period for the HOPU, which further demonstrated its utility in this setting. This also highlighted the value of repeated, longitudinal sessions so the providers can refresh their knowledge following gap in ultrasound use due to challenging circumstances. At the completion of the course, all participants demonstrated a sustained increase in PoCUS knowledge, confidence and experience. A local champion, better documentation, and practical ultrasound assessments for independent scanning would be the next steps in continuing the PoCUS education program at HOPU.

## Data Availability

The data used and analyzed during the current study are available from the corresponding author on reasonable request.

## Abbreviations

CT: Computed tomography
MRI: Magnetic resonance imaging
WHO: World Health Organization
PoCUS: Point-of-care ultrasound
US: United States
HOPU: Hospital Pediatrico Universitario
ED: Emergency department
PEM: Pediatric emergency medicine
EM: Emergency medicine
FAST: Focused assessment with sonography in trauma
